# Bacterial Diversity in Submarine Groundwater along the Coasts of the Yellow Sea

**DOI:** 10.3389/fmicb.2015.01519

**Published:** 2016-01-08

**Authors:** Qi Ye, Jianan Liu, Jinzhou Du, Jing Zhang

**Affiliations:** State Key Laboratory of Estuarine and Coastal Research, East China Normal UniversityShanghai, China

**Keywords:** Yellow Sea, submarine groundwater, bacteria, nutrient, bioremediation

## Abstract

Submarine groundwater (SGD) is one of the most significant pathways for the exchange of groundwater and/or source of nutrients, metals and carbon to the ocean, subsequently cause deleterious impacts on the coastal ecosystems. Microorganisms have been recognized as the important participators in the biogeochemical processes in the SGD. In this study, by utilizing 16S rRNA-based Illumina Miseq sequencing technology, we investigated bacterial diversity and distribution in both fresh well water and brackish recirculated porewater along the coasts in the Yellow Sea. The results showed that *Actinobacteria* and *Betaproteobacteria*, especially *Comamona*s spp. and *Limnohabitans* spp. were dominated in fresh well samples. Distinct patterns of bacterial communities were found among the porewater samples due to different locations, for examples, *Cyanbacteria* was the most abundant in the porewater samples far from the algal bloomed areas. The analysis of correlation between representative bacterial taxonomic groups and the contexture environmental parameters showed that fresh well water and brackish porewater might provide different nutrients to the coastal waters. Potential key bacterial groups such as *Comamonas* spp. may be excellent candidates for the bioremediation of the natural pollutants in the SGD. Our comprehensive understanding of bacterial diversity in the SGD along the coasts of the Yellow Sea will create a basis for designing the effective clean-up approach *in-situ*, and provide valuable information for the coastal management.

## Introduction

Submarine groundwater discharge (SGD) includes both fresh meteoric groundwater to the ocean by terrestrially-driven directly and recirculated seawater by permeable sediment (Garcia-Solsona et al., 2010). More and more works showed that SGD is one of the most important pathways for the exchange of groundwater and/or source of dissolved compounds (e.g., nutrients, metals, carbon) to the ocean, which may cause negative impacts on the coastal ecosystems (Burnett et al., 2006; Moore, 2010). Nitrogen input through the SGD may contribute to the eutrophication of coastal regions (Moore, 2010). Several case studies showed that increased nutrient supply via SGD might be a key factor for initiating and fueling the persistent harmful algal blooms (HABs) (LaRoche et al., 1997; Hu et al., 2006; Lee and Kim, 2007; Smith and Swarzenski, 2012).

The Yellow Sea, which is as a shallow semi-enclosed water body of the Northwest Pacific Ocean, shows such a strong seasonality in the nutrients concentration and their components (Liu et al., 2013). SGD has been determined as a main nutrient source to the Yellow Sea, which is one of the largest continental shelves in the world (Kim et al., 2005; Waska and Kim, 2011). Microorganisms have been shown as the important components mediating chemical reactions in the SGD in some concerned cases (Boehm et al., 2004; Paytan et al., 2004; Halliday and Gast, 2010). Recent studies focused on the bacterial communities in the surrounding sea water during macroalgal blooms of *Ulva prolifera* in the Yellow Sea (Burke et al., 2011; Liu et al., 2011; Zhang et al., 2014). For example, Analysis of *nifH* gene clone library revealed that heterotrophic nitrogen fixers, mostly represented by *Vibrio*-related *Gammaproteobacteria*, were dominated in both of surface waters that were covered and non-covered with massive macroalgal canopies of *U. prolifera* in the Yellow Sea in the summer of 2011 (Zhang et al., 2014). However, for our knowledge, up to now, there are few reports on the bacterial compositions in SGD along the coast of the Yellow Sea.

Microorganisms play pivotal roles in biogeochemical cycles in marine ecosystems (DeLong and Karl, 2005; Zehr and Kudela, 2011). It was reported that several studies using 16S rRNA (Fields et al., 2005), functional genes (Yan et al., 2003; Santoro et al., 2006, 2008), microarray (Waldron et al., 2009), and metagenomics approaches (Hemme et al., 2010) to reveal bacterial communities in groundwater systems. The decreased diversity was observed in groundwater contaminated with higher levels of nitric acid and uranium waste in the Field Research Center (FRC) of the U.S. Department of Energy Environmental Remediation Science Program (Oak Ridge, TN, USA) (Fields et al., 2005). Ammonia-oxidizing bacterial and archaeal abundance as well as denitrifier communities were characterized along a nitrate and salinity gradient in groundwater samples at Huntington Beach, CA, USA (Santoro et al., 2006, 2008). Metagenomical analysis showed that a groundwater microbial community composed of clonal denitrifying γ- and β-proteobacterial populations in an extreme low-pH environment contaminated with high levels of uranium, nitric acid, technetium and organic solvents (Hemme et al., 2010). Biodegradation or biotransformation of pollutants with microorganisms is the most potential cost-effective bioremediation technology in the natural environments (Fields et al., 2005; Hwang et al., 2009). Pilot-scale uranium *in situ* bioremediation experiment suggested that the indigenous microbial communities can be successfully stimulated after 2 years of biostimulation with ethanol in FRC groundwater ecosystem, U(VI) levels were reduced from the initial concentration of 50 mg/L to below drinking water standard (< 30 μg/L) (Xu et al., 2010). Zhang et al. (2015) reported that amendment of the slow-release polylactate hydrogen-release compound (HRC) stimulated FRC groundwater microbial communities associated with HRC degradation and reduction of ${\text{NO}}_{3}^{-}$, Cr(VI), Fe(III), and ${\text{SO}}_{4}^{2-}$. In the present work, by using 16S rRNA gene-based Miseq Illumina sequencing approach, we focused on the bacterial compositions in both fresh well water and recirculated brackish porewater along the coast of the Yellow Sea. The goal is to explore the key bacterial groups, which can play their potential ecological roles in nitrogen and carbon flux by SGD to the coastal areas. The relationship between representative taxonomic groups and contexture environmental parameters were also investigated. Our understanding on the potential bacterial candidates for bioremediation will help us design the effective clean-up technology for coastal environmental management.

## Materials and methods

### Sample collection and measurements of physic-chemical parameters

Groundwater samples (well and pore water) in our field observation were collected from wells and beach in May, 2014. Well samples along the shore were pumped from the wells utilizing the water pump; pore water samples from offshore zone were collected from a push-point piezometer using a peristaltic pump (Charette and Allen, 2006). The temperature, salinity and pH of groundwater were measured directly in the field using a portable salinometer with multiple parameters (Germany, multi 350i). Sampling depth of each sample site ranged from 0.4 m to > 20 m (Table 1).

**Table 1:** **Site descriptions and chemical measurement**.

**Sample name**	**YSGW^a^-1**	**YSGW-3**	**YSGW-4**	**YSGW-11**	**YSPW^b^-2**	**YSPW-7**	**YSPW-11**
Latitude	32°25'41.6″	35°38'43.5″	36°03'45.0″	37°07'32.7″	35°25'28.8″	37°34'26.4″	37°31'33.0″
Longitude	121°17'43.8″	119°54'38.6″	120°20'39.1″	122°27'47.2″	119°34'00.5″	121°15'10.6″	122°01'46.9″
Location	Nantong City, Jiangsu Province	Qingdao, Shandong Province	Qingdao, Shandong Province	Weihai, Shandong Province	Rizhao, Shandong Province	Yantai, Shandong Province	Weihai, Shandong Province
Type of groundwater or porewater^*^	Pore water in unconsolidated deposits	Bedrock fissure water	Bedrock fissure water	Bedrock fissure water.	In the beach with coarse sand	In the beach with coarse sand	In the beach with coarse sand
Characteristics of sampling site	Residential area; mainly fishery industry around	Near the village; In the open air; Abandoned well	In the park; In the open air; Abandoned well;garbage found	Residential area; Domestic water, but not for drinking	A long and big tourist beach with gentle slope near a wharf,	Tourist beach with gentle slope	Tourist beach
Salinity	1.4	0.3	0.4	0.4	29.6	29.5	29.8
Temperature	17.2	15.3	14.4	14.5	16.4	15.5	14.5
pH	7.883	7.514	8.044	7.882	7.945	7.802	7.942
Sampling depth (m)	~15	>20	1.0	6.8	1.3	0.4	0.55
^224^Ra(dpm/L)	0.222	0.379	0.181	0.291	6.493	7.556	6.367
^223^Ra(dpm/L)	0	0.013	0.014	0.0062	0.094	0.193	0.145
${\text{NO}}_{3}^{-}$(μmol/L)	243.9	442.7	20.4	540.1	7.7	1.6	14.8
${\text{NO}}_{2}^{-}$(μmol/L)	0.41	0.24	0.50	0.45	0.98	0.24	0.13
${\text{NH}}_{4}^{+}$(μmol/L)	1.55	1.19	9.56	0.98	8.09	19.40	13.27
${\text{PO}}_{4}^{3-}$(μmol/L)	7.19	0.78	0.21	0.79	0.62	0.75	0.89
${\text{SiO}}_{3}^{2-}$(μmol/L)	333.3	248.5	189.8	116.2	4.5	13.3	5.6
DOC^c^(μmol/L)	141	83	332	201	165	99	76

a *Well water*.

b *Pore water*.

c *DOC, Dissolved Organic Carbon*.

* *Based on the hydrogeological map of China: China Cartographic Publishing House, prepared by the Institute of Hydrogeology and Environment Geology, Chinese Academy of Geological Science, 1984*.

Four well water samples (~40 L) and three pore water samples (~20 L) were collected for Ra isotope measurement. After filtrating (pore size: 0.5 μm), Ra isotopes in the water were extracted using a MnO_2_-impregnated acrylic fiber column (20 g), then the column with Mn-fiber was immediately placed in the Radium Delayed Coincidence Counter (RaDeCC) to measure the short-lived isotope ^223^Ra and ^224^Ra in the field (Moore and Arnold, 1996).

Corresponding water samples (~60 mL) for nutrient analysis were collected with polyethylene bottles filtered through 0.45 μm cellulose acetate filters. Then the filtrates were poisoned with saturated HgCl_2_ and stored in the dark. The nutrient concentrations (${\text{NO}}_{2}^{-}$, ${\text{NO}}_{3}^{-}$, ${\text{NH}}_{4}^{+}$, ${\text{PO}}_{4}^{3-}$, and ${\text{SiO}}_{3}^{2-}$) were then analyzed using an auto-analyzer (Model: Skalar SANplus146) (Liu et al., 2005). DOC samples were filtered via clean Nylon filter (pore size: 0.45 μM) immediately after collection and kept at −20°C until analysis. In the lab, DOC samples were measured with a TOC analyzer (Shimadzu® TOC-LCPH).

Replicate, 300-mL of each submarine groundwater sample were collected on a 0.22 μm pore size polycarbonate filter (Nuclepore Track-Etched Membrane, Whatman). The filter was placed in a sterile 1.5-mL microcentrifuge tube and immediately store at −20°C.

### DNA extraction, PCR, and sequencing

Total DNA was extracted from the filter using a MoBio PowerWater® DNA Isolation Kit (MOBIO Laboratories, Carlsbad, CA, USA) according to the manufacturer's instruction. DNA from two independent extractions were combined, and their concentration and purity were measured spectrophotometrically with NanoDrop ND2000.

Minimum numbers of PCR cycles were performed and three independent PCR mixtures were pooled for each sample to decrease PCR bias. Briefly, bacterial V4-V5 hypervariable regions of 16S rRNA genes were then amplified using the specific barcoded universal primer pairs 515F (5′-GTGCCAGCMGCCGCGG-3′) and 907R (5′-CCGTCAATTCMTTTRAGTTT-3′) (Xiong et al., 2012). Cycling conditions were 2 min at 95°C, followed by 25 cycles with 30 s at 95°C, 30 s at 55°C, and 30 s at 72°C, and a final extension period of 5 min at 72°C.

PCR products were purified using the AxyPreDNA gel extraction kit (Axygen Biosciences, USA) following the manufacturer's protocol and then quantified by QuantiFluorTM-ST (Promega, USA). Reaction mixtures were pooled in equimolar ratios and paired-end reads were generated on an Ilumina Miseq PE250 (Majorbio Bio-Pharm Technology Co., Ltd., Shanghai, China).

### Sequence data process, OTU cluster, and taxonomic assignment

Raw fastq files were demultiplexed, quality-filtered using QIIME (version 1.17) (Caporaso et al., 2010) with the criteria as described previously (Li et al., 2014). Read data of each sample was imported in Fastq format according to index barcode sequence. Operational Taxonomic Units (OTUs) were clustered at 97% similarity using UPARSE (version 7.1 http://drive5.com/uparse/). Chimeric sequences were identified and removed using UCHIME. By using the usearch_global command, the number of reads from each sample assigned to each OTU was generated in an “OTU table.” Representative 16S rRNA gene sequence of each OTU was analyzed by RDP Classifier (http://rdp.cme.msu.edu/) against the Silva (SSU115) 16S rRNA database with 70% confidence threshold.

### Phylogenetic analyses

The sequences of the representative OTUs obtained in this study were compared to those in the National Center for Biotechnology Information nucleotide database by using BLAST searching. The closest sequences and selected reference sequences were downloaded and aligned using Clustal W. Phylogenetic tree was created in MEGA6 using the neighbor-joining algorithm with a bootstrap test of 1000 replicates and maximum composite likelihood model (Tamura et al., 2013).

### Statistical analyses

Alpha diversity metrics and coverage were calculated using the Mothur Program (Schloss et al., 2009). R program was used to plot Heatmap figure (R Development Core Team., 2013). Bray-Curtis dissimilarities were calculated based on genus/sample abundance matrix using function vegdist of the vegan package in R (Oksanen et al., 2007). Hierarchical clustering of the samples was performed with the complete method using the function hclust of stats package in R (R Development Core Team., 2013).

Pearson's correlation coefficient was calculated using SPSS software, corresponding heatmap was plotted using gplots package in R (Oksanen et al., 2007; Warnes et al., 2012).

### Nucleotide sequence accession numbers

Paired end Illumina sequence data from this study were submitted to the NCBI Sequence Read Archive (SRA) under accession number SRP064231.

## Results

### Site description and physic-chemical characteristics

Fresh well water and brackish porewater of submarine groundwater samples were selected in this study. Wells along the shore were dug by native habitants; Porewater within Yellow Sea offshore area were collected from ~50 to 130 cm below the sediment-water interface (Table 1).

**Figure 1:**
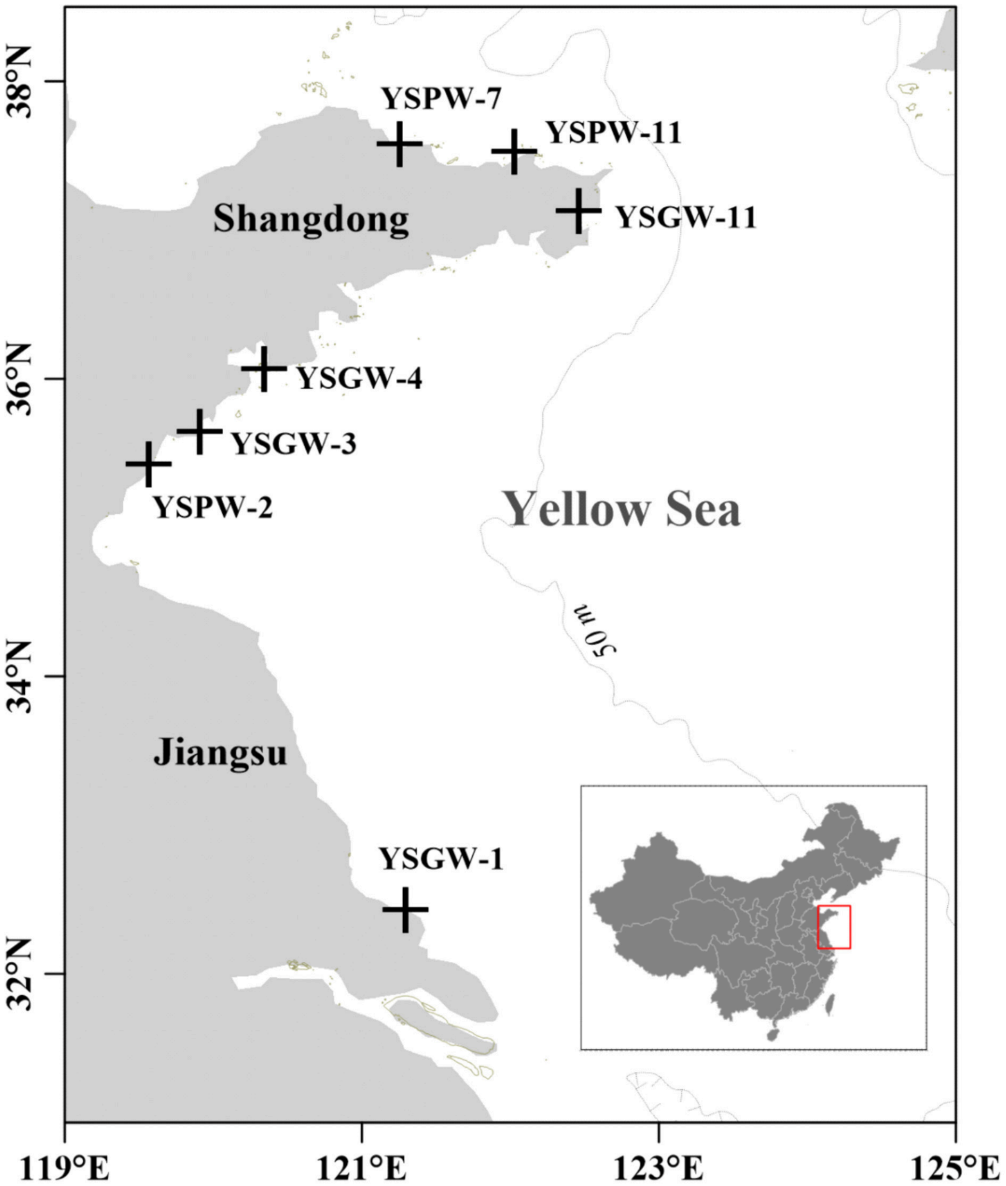
**Map showing the sampling stations of submarine groundwater along the coasts of the Yellow Sea**.

Based on the types of submarine groundwater and physicochemical parameters, seven samples were separated into two groups: Group W included four samples (YSGW1, YSGW3, YSGW4, and YSGW11) collected from the wells, the salinity of well waters ranged from 0.3 to 1.4‰, indicating their freshwater environment; Group P included three porewater samples (YSPW2, YSPW7, and YSPW11) with relative higher salinity (~30‰) (Table 1, Figure 1).

The other hydrological properties and chemical parameters of submarine groundwater are also summarized in Table 1. Fresh well waters had an average value of 0.269 and 0.0083 dpm/L for ^224^Ra and ^223^Ra. However, pore water had a higher value for ^224^Ra and ^223^Ra, was 6.805 and 0.144 dpm/L, respectively. Several nutrients in the specific well water samples were much higher than those in the pore water, for examples, nitrate concentrations ranged from 243.9 to 540.1 μmol/L within Group W except sample YSGW 4 (20.4 μmol/L), but still higher than those in porewater samples (1.6–14.8 μmol/L). Ammonium concentration ranged 8.09–19.40 μmol/L in porewater samples, and 0.98–1.55 μmol/L within Group W except sample YSGW 4 (9.56 μmol/L). Porewater sample YSGW 2 had the highest nitrite concentration and well water sample YSGW 1 had the highest phosphate concentration. The silicate concentrations in porewater samples were below 15 μmol/L, while the concentrations in well samples were much higher (116.2–333.3 μmol/L). No distinct pattern of DOC concentrations between well water and porewater samples, but YSGW 4 had the highest DOC concentration. The other parameters such as pH and temperature were relatively constant at all sites.

### Bacterial diversity and cluster analysis

We obtained a total of 87,475 high quality bacterial V4-V5 Illumina sequences, with an average read length of 396 bp from seven submarine groundwater samples.

According to 97% identity cutoff, there were 1078 OTUs in the complete data set. Among them, only 23 OTUs were found in all well water samples and 169 OTUs in porewater samples. Good's coverage was 99~99.8% for all samples, representing almost whole range of bacterial diversity. The Chao, ACE value, and Shannon evenness all indicated that the α-diversity of bacterial community is lower in the well water samples than in porewater samples (Table 2).

**Table 2:** **Diversity and richness estimators for Illumina libraries**.

**Station**	**Optimized sequence #**	**Observed OTU**	**Unique OTU**	**Chao^a^**	**ACE^b^**	**Shannon index**	**Coverage (%)**
YSGW1	13689	249	105	330	297	2.83	99.6
YSGW3	10964	184	48	202	209	2.82	99.7
YSGW4	12911	69	10	103	113	2.81	99.8
YSGW11	16513	151	21	177	184	1.98	99.8
YSPW2	7499	505	152	562	546	5.35	99
YSPW7	11047	364	54	456	454	3.27	99.1
YSPW11	14852	548	143	584	584	4.56	99.5

a *Chao 1 species richness*.

b *Abundance-based coverage estimator*.

**Figure 2:**
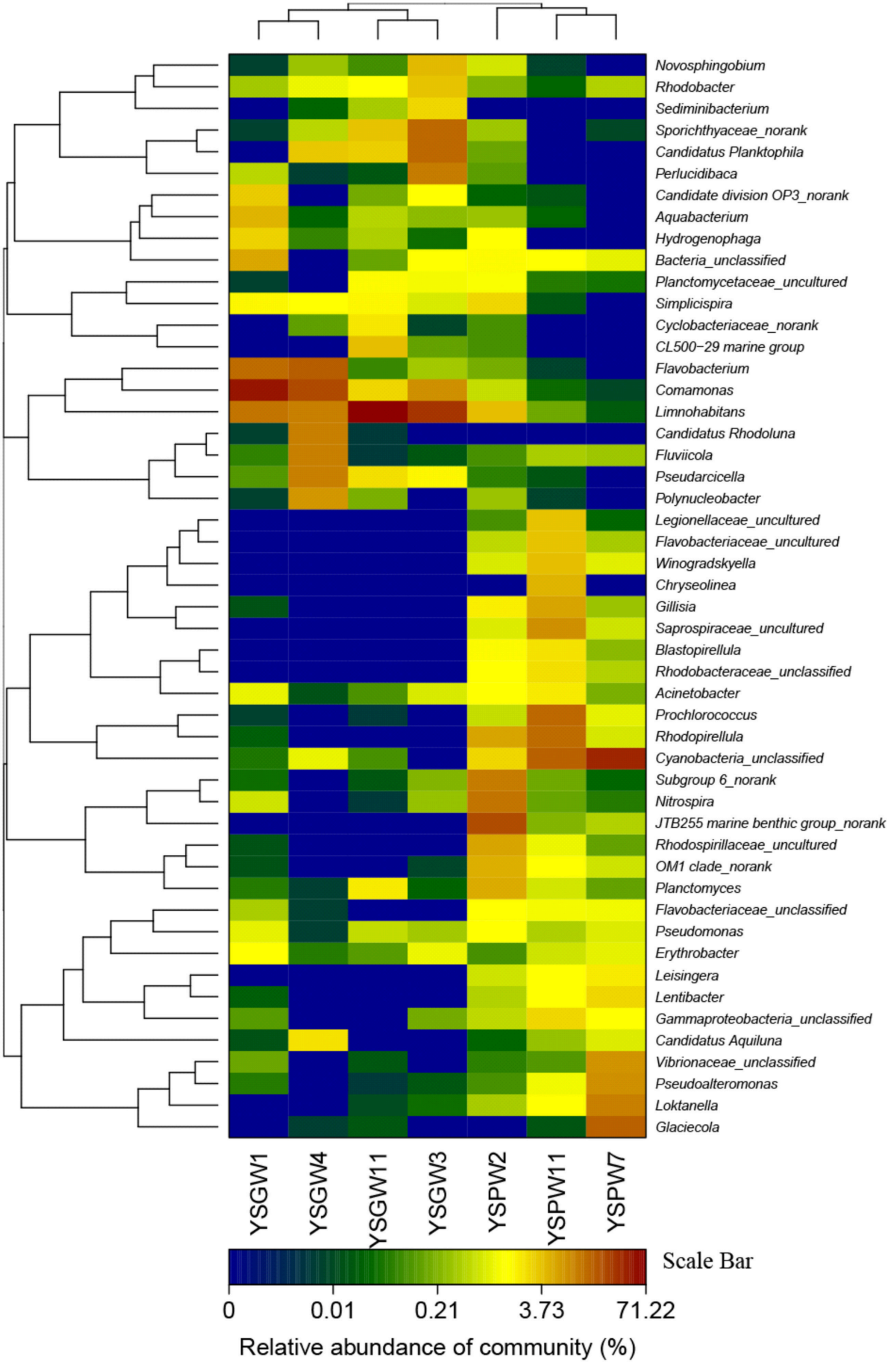
**Heatmap showing the relative abundance and distribution of genus-based OTU illumina reads**. The color code indicates relative abundance, ranging from blue (low abundance) to black to brown (high abundance).

The hierarchical heatmap at the bacterial genus level showed that seven samples could be organized into two main groups: The first group was composed of four well water samples, YSGW1 and YSGW4 were clustered together, and YSGW3 and YSGW11 formed another cluster; the second group was composed three porewater samples, turbid porewater YSPW2 was clustered separately from clear porewater YSPW7 and YSPW11 (Figure 2).

### Bacterial distribution

**Figure 3:**
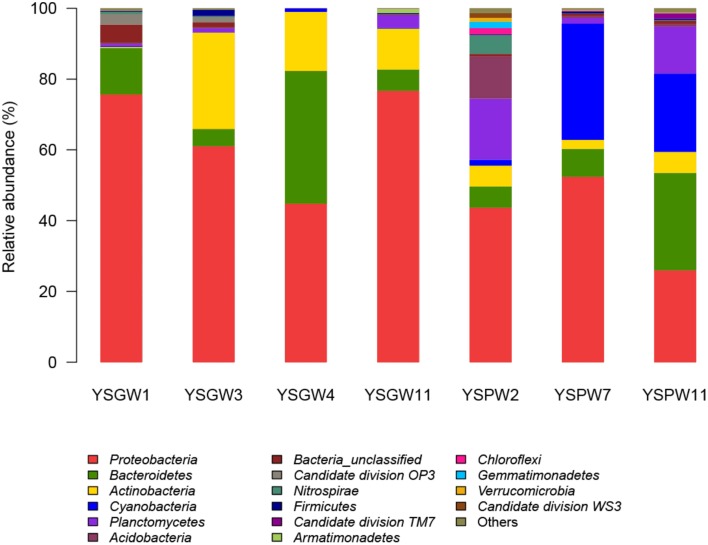
**Phylum-level taxonomic distribution**. Bars represented the percentage of Illumina tag composition represented by each phylum. Bacterial taxa represented by less than 1% reads are pooled as “other.”

Figure 3 showed that there were some variations in the percentage composition of illumina sequences among the samples. *Proteobacteria* was the most abundant phylum in all samples (43.7~76.7%) except YSPW11 (25.9%). *Bacteriodetes*, which was the most abundant phylum in YSPW11 (27.5%), was also distributed in the other samples. *Actinobacteria* was the other prevalent phylum in wellwater samples YSGW3 (27.2%), YSGW4 (16.7%), and YSGW11 (11.6%). *Planctomycetes* and *Acidobacteria* formed the second (17.2%) and third (12%) dominant groups after *Proteobacteria* within YSPW2. *Cyanobacteria* represented 32.8 and 22.7% of sequences within porewater samples YSGW7 and YSGW11. Interestingly, *Cyanobacteria* was almost absent from the other five samples.

### Phylogenetic analyses

#### Main groups of *Actinobacteria* and *Betaproteobacteria* within Group W samples

**Figure 4:**
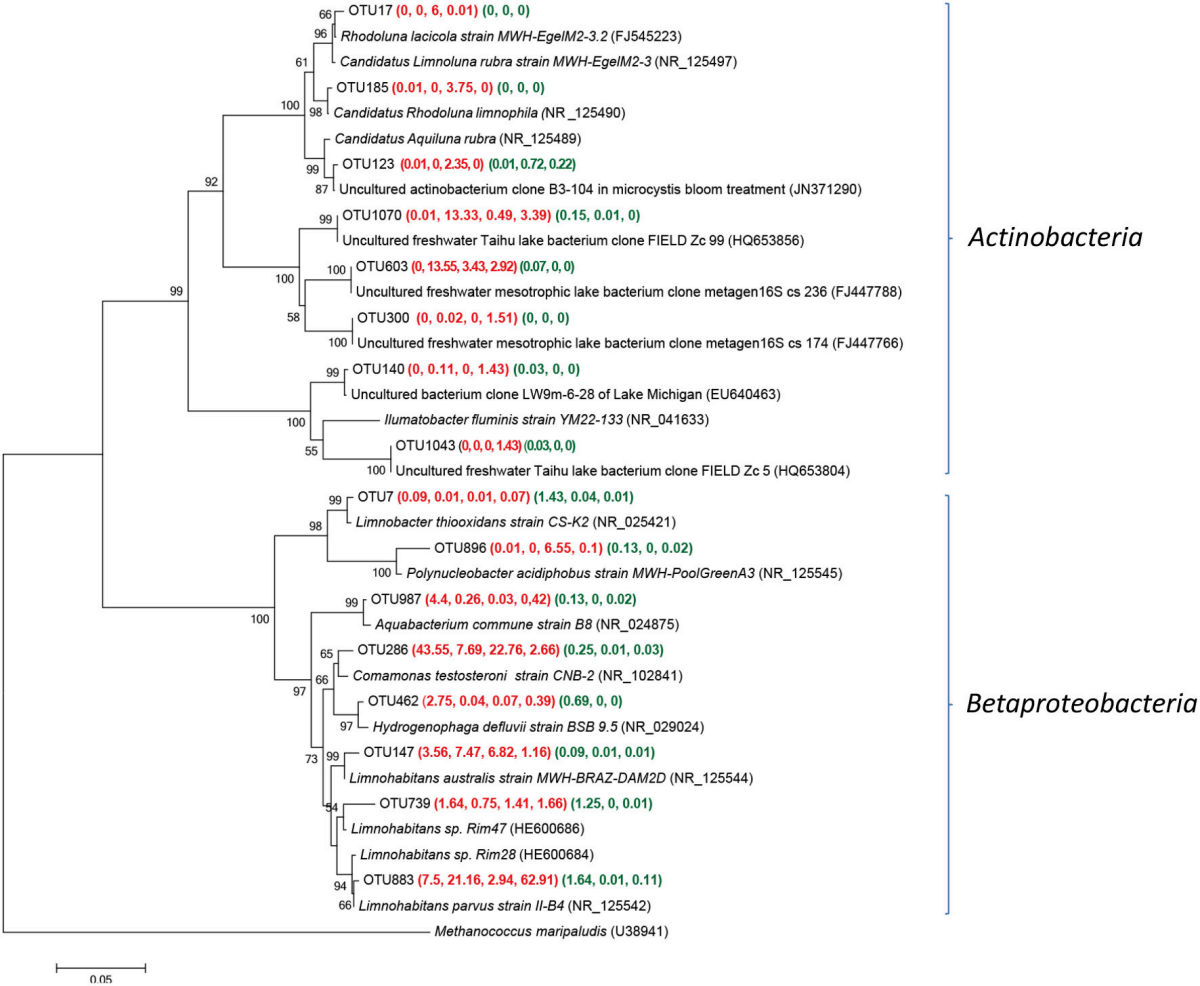
**Neighbor-joining tree showing phylogenetic relationships among the representative OTUs obtained in this study and reference 16S rRNA sequences retrieved from the NCBI GenBank for ***Actinobacteria*** and ***Betaproteobacteria*****. These OTUs were represented by more than 1% reads from a single station and/or from multiple stations within well water samples. The OTUs obtained in this study are shown in bold type. The numbers in parentheses indicate the percentage composition of reads in each station in the following order: **(YSGW1, YSGW3, YSGW4, YSGW11) (YSPW2, YWPW7, YSPW11)**. The scale bar represents the estimated number of nucleotide changes per sequence position. The numbers at the nodes show the bootstrap values obtained after 1000 resamplings, only values of > 50 are shown. Accession numbers for the reference sequences are shown in parentheses.

OTUs related to *Actinobacteria* and *Bataproteobacteria* were frequently detected within fresh well water samples (Figure 4). The *actinobacterial* sequences of OTU 1070 and 603 was the most frequently retrieved from YSGW3, which were identical to the environmental clones from Taihu (China) (HQ653856) (Zeng et al., 2012a) and Bourget (French) (FJ447766) (Debroas et al., 2009). Sequences of three OTUs 17, 123, and 185 were obtained from YSGW4, had 99.5, 99.7, 99.0% similarity with three representative planktonic freshwater *Actinobacteria Rhodoluna lacicola* strain MWH-EgelM2-3.2 (FJ545223) (Sharma et al., 2009), Candidatus *Rhodoluna limnophila* (NR_125490) (Hahn, 2009), Candidatus *Aquiluna rubra* (NR_125489) (Hahn et al., 2004) respectively (Figure 4).

Class *Betaproteobacteria* accounted for 38.7–70.5% within Group W, and there is a niche separation among different genus within *Betaproteobaceria* (Figure 4). Both YSGW1 and YSGW4 were characterized by codominance of genera *Comamona*s (43.6 and 22.8%), and *Limnohabitans* (11.1 and 9.8%), *Limnohabitans* was the most abundant genus in YSGW3 (28.6%) and YSGW11 (64.1%). Representative OTU286 had 99% similarity with *Comamonas testosteroni* strain CNB-2 (NR_102841), which is capable of degrading aromatic compounds (Ma et al., 2009), and OTU 883 had 99.5% similarity with nitrogen cycling *Betaproteobacteria Limnohabitans* sp. Rim28 (HE600684) (Kasalickı et al., 2013) and 99.7% similarity with *Limnohabitans parvus* strain II-B4 (NR_125542). OTU 896 accounted for 6.55% of total bacteria in YSGW4, but seldom detected in other samples, had 99% similarity with *Polynucleobacter acidiphobus* strain MWH-PoolGreenA3 (NR_125545), which may utilize autochthonous substrate sources primarily supplied by phytoplankton (Hahn et al., 2011).

#### Main groups of *Gammaproteobacteria, Cynobacteria*, and *Planctomycetes* within Group P samples

**Figure 5:**
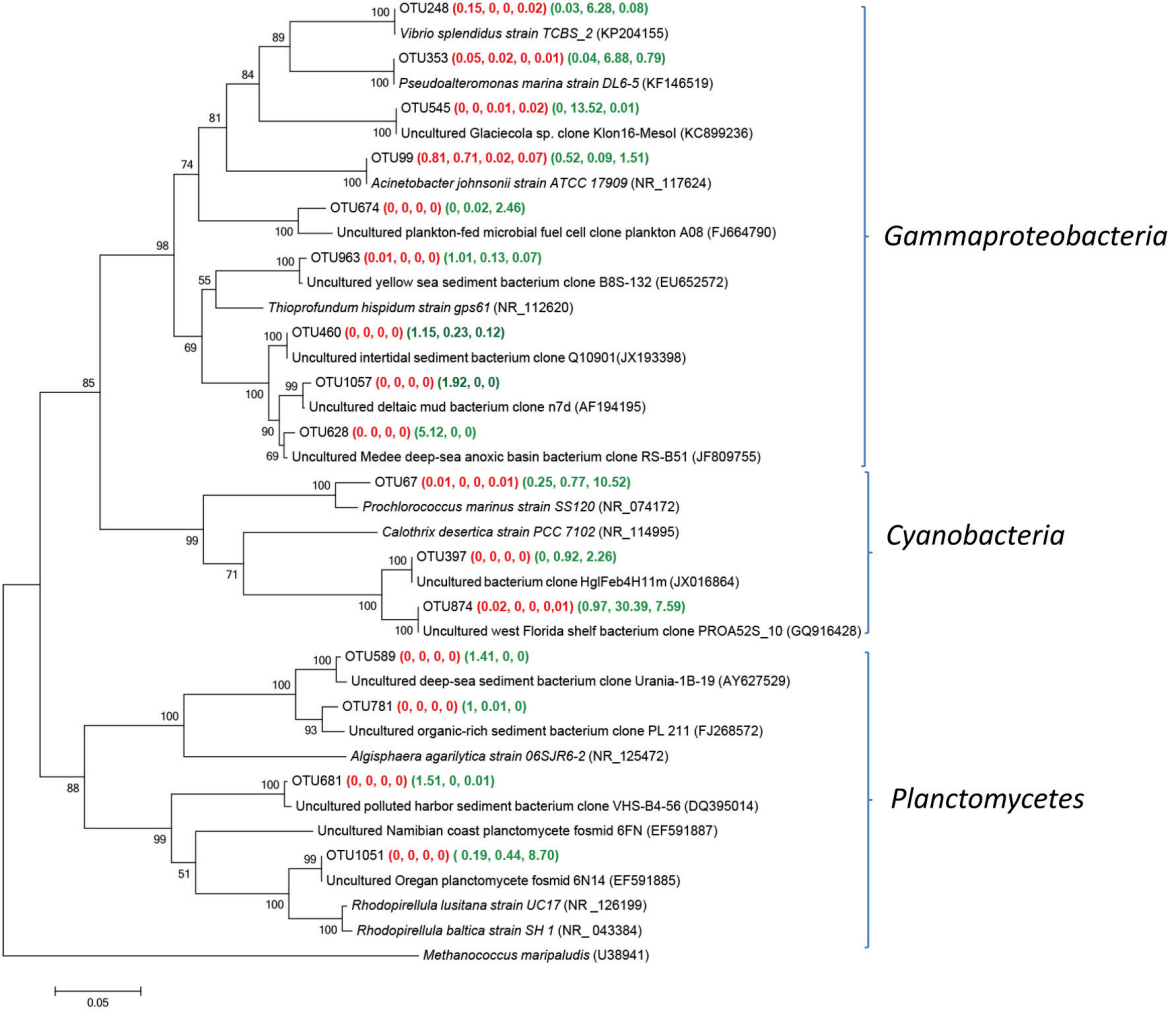
**Neighbor-joining tree showing phylogenetic relationships among the representative OTUs obtained in this study and reference 16S rRNA sequences retrieved from the NCBI GenBank for ***Gammaproteobacteria***, ***Cyanobacteria***, and ***Planctomycetes*****. These OTUs were represented by more than 1% reads from a single station and/or from multiple stations within porewater samples. The OTUs obtained in this study are shown in bold type. The numbers in parentheses indicate the percentage composition of reads in each station in the following order: **(YSGW1, YSGW3, YSGW4, YSGW11) (YSPW2, YWPW7, YSPW11)**. The scale bar represents the estimated number of nucleotide changes per sequence position. The numbers at the nodes show the bootstrap values obtained after 1000 resamplings, only values of > 50 are shown. Accession numbers for the reference sequences are shown in parentheses.

*Gammaproteobacteria, Cyanobacteria* or *Planctomycetes* was dominant in one or two Group P samples (Figure 5). The sequences of OTU 874, representing 30.4 and 7.6% in YSPW7 and YSPW11 respectively, were remotely related to the cyanobacterium *Calothrix desertica* strain PCC 7102 (NR_114995) (86% similarity) (Sihvonen et al., 2007). OTU 874 was also associated phylogenetically (100% similarity) with a cyanobacterial clone PROA52S_10 (GQ916428) recovered from the Florida west surface coastal water with high cell abundance of brevetoxin-producing dinoflagellate *Karenia brevis* (Jones et al., 2010). In addition, OTU 67 containing 12.5% sequences in YSGW11, had 97% similarity with *Prochlorococcus marinu* SS120 (NR_074172) with a nearly minimal oxyphototrophic genome (Dufresne et al., 2003).

OTU 248, 353, and 545 gave the 100% match to *Gammaproteobacteria Vibrio splendidus* strain TCBS_2 (KP204155), *Pseudoalteromonas marina* strain DL6-5 (KF146519) (Wang et al., 2014), and Uncultured *Glaciecola* sp. clone Klon16-MesoI (KC899236) (Scheibner et al., 2014). Sequences of these three OTUs accounted for 26.68% of total bacteria in YSGW7. Within class *Gammaproteobacteria*, we found OTU628 matched sedimentary clone from the Medee deep-sea hypersaline anoxic basin (JF809755) (Akoumianaki et al., 2012) at 99.2% similarity and has its highest relative abundance in YSPW2 (Figure 5).

Approximately 8.7% sequences of YSPW11 were grouped into OTU1051, showed 100% identity with fosmid 6N14 (EF591885), which was retrieved from 200 m depth off the coast of Oregon (Woebken et al., 2007), they were also related to *Rhodopirellula baltica* strain SH 1^T^ (NR_043384) encoding high numbers of sulfatases (~95% similarity) (Glöckner et al., 2003).

#### Main groups of *Bacteriodetes* detected in both Group W and P samples

**Figure 6:**
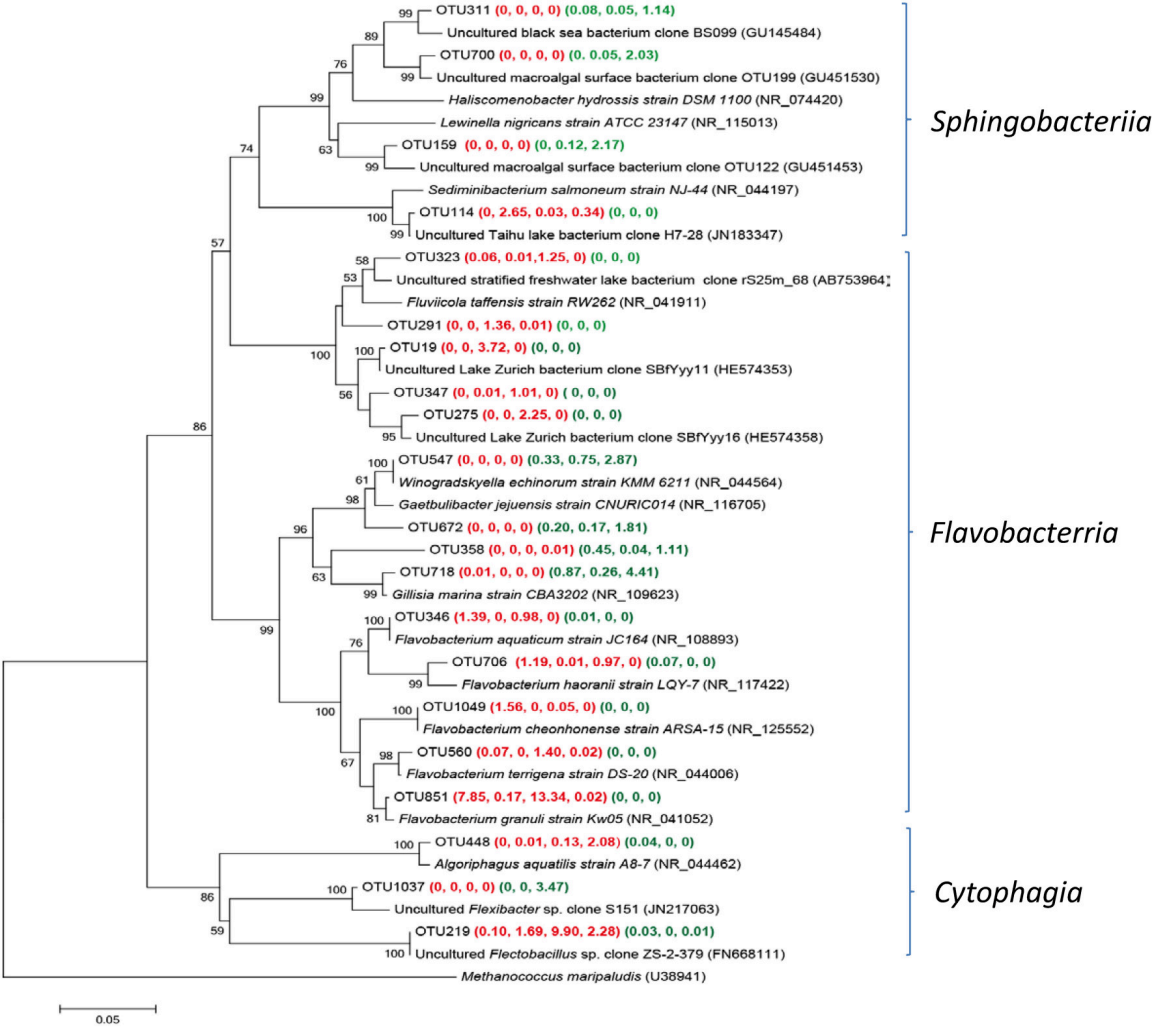
**Neighbor-joining tree showing phylogenetic relationships among the representative OTUs obtained in this study and reference 16S rRNA sequences retrieved from the NCBI GenBank for ***Bacteriotedes*****. These OTUs were represented by more than 1% reads from a single station and/or from multiple stations within well water or porewater samples. The OTUs obtained in this study are shown in bold type. The numbers in parentheses indicate the percentage composition of reads in each station in the following order: **(YSGW1, YSGW3, YSGW4, YSGW11) (YSPW2, YWPW7, YSPW11)**. The scale bar represents the estimated number of nucleotide changes per sequence position. The numbers at the nodes show the bootstrap values obtained after 1000 resamplings, only values of >50 are shown. Accession numbers for the reference sequences are shown in parentheses.

*Bacteriodetes* was frequently observed in all studied submarine groundwater samples, however, the main groups of *Bacteroidetes* were different between Group W and P samples (Figure 6). Five *Flavabacterium* related OTUs were found in YSGW1 and YSGW4, indicating microbial degradation of high-molecular-weight organic matter in these samples (Kirchman, 2002). Two OTUs 19 and 275 in YSGW4 matched uncultured environmental clones from Lake Zurich (HE574353 and HE574358), which may participate in N-acetyl-glucosamine uptake (Eckert et al., 2012). OTU 219 had a significantly higher abundance in the YSGW4 and 100% identity to uncultured *Flectobacillus* clone ZS-2-379 (FN668111) (Van den Wyngaert et al., 2011), members of the grazing-resistant *Flectobacillus* genus had the ability to defense filament-forming predators (Šimek et al., 2010a). OTUs 159 and 700 in YSGW11 were closely affiliated with clones recovered from macroalgal surface (GU451453 and GU451530) (Lachnit et al., 2011).

### Correlation analysis between bacterial taxonomic groups and environmental parameters

**Figure 7:**
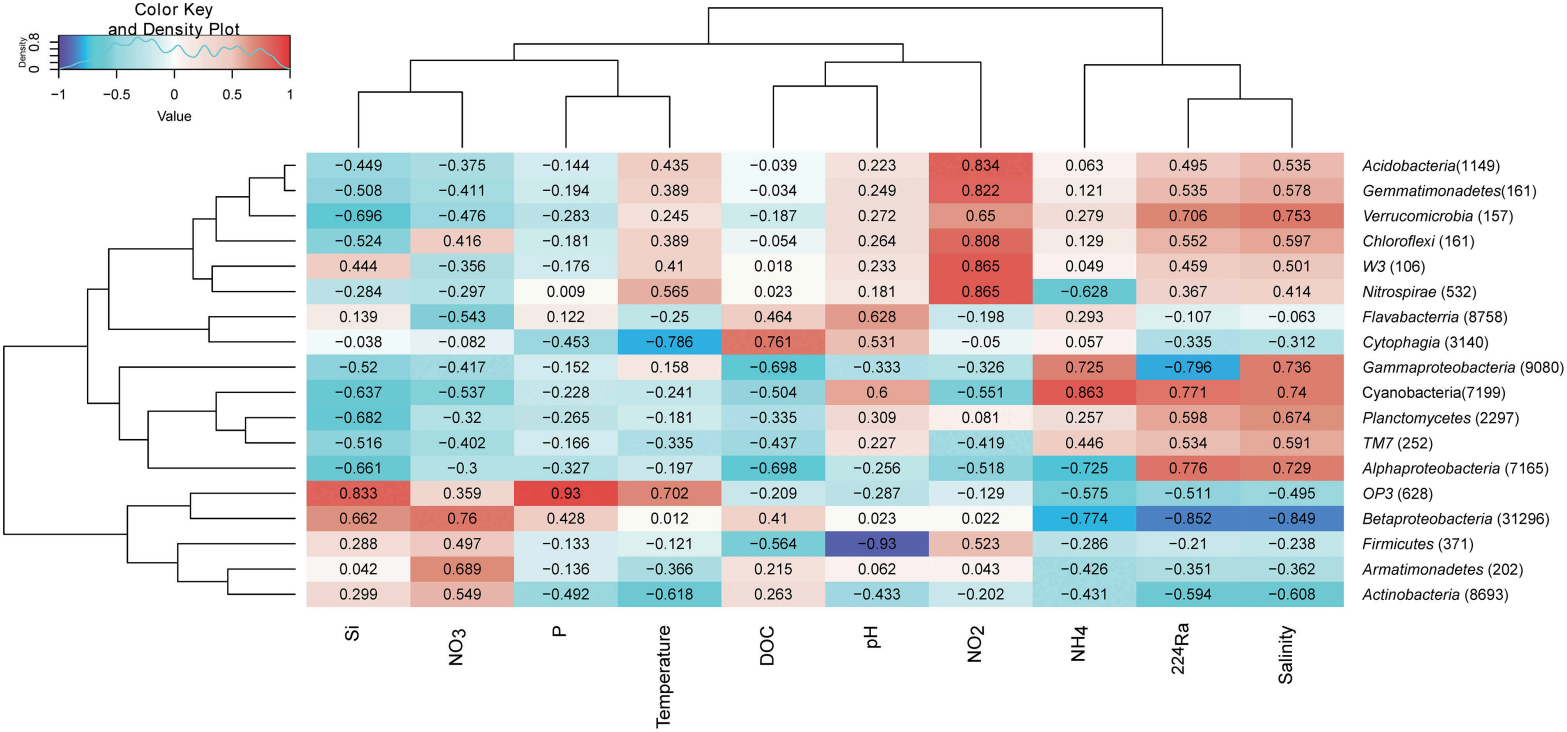
**Environmental factors associated with variations of the bacterial community structure at the phylum level**. In order to obtain a higher resolution, the *Proteobacteria* phylum level was separated into *Alpha, Beta*, and *Gamma-Proteobacteria* classes. Phylum *Bacteriodetes* was separated into classes *Flavabacterria* and *Cytophagia*. The total number of sequences in each phylum is indicated in parentheses. Pearson's correlation coefficients between -1 and 1 are shown in the rectangle, which indicates correlations between phylum/class sequence abundance and selected environmental parameters. For example, a firebrick colored rectangle (0.76) between nitrate and *Betaproteobacteria* indicates a higher number of sequences with increasing nitrate concentration, a blue rectangle (−0.774) between *Betaproteobacteria* and ammonium indicates a higher number of sequences with decreasing ammonium concentration. The color code indicates Pearson's correlation coefficients, ranging from blue (−1) to white (0) to firebrick (1). The density showed the distribution of Pearson's correlation coefficients between −1 and 1. Si, silicate; P, phosphate; NO_2_, nitrite; NO_3_, nitrate; NH_4_, ammonium; ^224^Ra, Radium isotope tracer; DOC, dissolved organic carbon.

In order to investigate whether some bacterial taxonomic groups are characteristics of specific environmental conditions, we calculated the Pearson's correlation coefficient between bacterial taxonomic groups and contextual environmental parameters (Gobet et al., 2012). The selected phyla/classes were divided into two groups: rare taxonomic group is defined as the phylum/class represented by more than 1% reads only from a single station, whereas abundant taxonomic groups is defined as the phylum/class represented by more than 1% reads from at least two stations (Table S1). Within abundant taxonomic groups, we found significant Pearson's correlation coefficients between isotope ^224^Ra tracers and Phyla including *Cyanobacteria, Betaproteobacteria*, and *Gammaproteobacteria*. Salinity is an important environmental parameter which significantly influenced the *Betaproteobacteria* (−0.849). Pearson's correlation coefficient between *Betaproteobacteria* and nitrate (0.76)/ammonium (−0.774) suggested increased in sequences as nitrate concentration got higher and ammonium concentration got lower, indicating ammonium oxidation by *Betaproteobacteria*. *Cyanobacteria* was positively correlated with the ammonium concentration (0.863) suggesting nitrogen fixation by *Cyanobacteria*. Sequences within class *Cytophagia* had highly negative correlation with temperature but positive with DOC concentration, suggested organic matter degradation of this low temperature favorable bacterial group. Interestingly, six out of nine rare taxonomic groups were detected in porewater YSPW2, and these six groups had highly positive correlation with nitrite concentration (Figure 7).

## Discussion

### Distinct bacterial communities within porewater samples

In this study, different locations may result in distinct bacterial communities among the porewater samples, for example, YSPW 2 was close to the regions where the green tides frequently break out, and there were specific bacterial taxonomic groups only detected in this site, but lacked some phyla which were abundant in other two porewater samples (Figure 5). One of the negative effects of the green tides on the marine ecosystem was the production of toxic hydrogen sulfide into surrounding environments (Lyons et al., 2014). The second most sequences in turbid YSPW 2 porewater sample were affiliated with JTB255 cluster, which was considered as the putative sulfide-oxidizing bacteria (Bowman and McCuaig, 2003). It is proposed that the toxic hydrogen sulfide could be detoxified in the porewater.

The *Cyanobacteria* was the most abundant group with OTU 874 comprising 30.4% in YSPW 7 and 7.6% in YSPW11, but only 0.97% in YSGW 2. Sequences of OTU 874 were remotely related to the heterocystous diazotrophic cyanobacterium *Calothrix* genus (Zehr, 2011), and 100% identical to the environmental clone PROA52S 10 from *Karenia brevis* dominated surface water (Jones et al., 2010), showing this uncultured cyanobacterial group might pose the putative ecological functions to different phytoplankton blooms. In addition, the most abundant OTU 67 in YSGW 11, had 97% similarity with the non-diazotrophic picocyanobacterium *Prochlorococcus marinus* strain SS12, suggesting the cohabitation of two groups of cyanobacteria in this sample. Unicellular phototroph *Prochlorococcus marinus* strain SS12 is well known for its characteristics of the tiny cell size, autotrophic metabolism and adaption to low light niche (Dufresne et al., 2003), this low-light-adapted ecotype may make it possible to survive in the porewater at dimmer light.

It has been shown that microalgal growth was linked to nutrients released by the nitrogen fixing cyanobacteria. For instance, following aerolian iron input, nitrogen fixer *Trichodesmium* produced dissolved organic nitrogen (DON) for stimulating the growth of *Karenia brevis*, subsequently caused the dinoflagellate bloom in the Gulf of Mexico waters (Lenes et al., 2001). Jones et al. (2010) found the higher abundance of cyanobacteria in the zero/low *K. brevis* libraries than those of medium/high K. brevis libraries. The *Cyanobacteria* was also detected in the surrounding water during an early *Ulva* bloom (Liu et al., 2011). Notably, cyanobacteria usually grow better at the early stage of macroalgae blooms, this could be explained that allelopathic chemicals produced by periphyton biofilms suppress cyanobacterial growth in macroalgae dominated waters (Wu et al., 2011). This may explained lower abundant cyanobacteria detected in YSPW 2. We speculated that the newly fixed N released by the *Cyanobacteria* in surface water and porewater might be significant in initiating the algal blooms. Considering high abundance of cyanobacteria detected in porewater samples, SGD became the potential nutrient source for adjacent coastal areas.

Bacterial community structures associated with the surface of *Ulva prolifera* and in surrounding seawater in Jiaozhou Bay during the *Ulva* bloom phases in 2008 were reported (Liu et al., 2011). They found Orders *Alteromonadales, Flavobacteriales*, and *Rhodobacterales* were the common bacterial groups on the surface of *U. prolifera*, and *Alteromonaldales* was also dominant in the seawater. The genus *Glaciecola* within *Alteromonadales* was dominated not only in the algal surface but also in the seawater. In porewater sample YSGW7, sequences affiliated with three orders accounted for 42.7% of total bacteria. Ra isotope analysis showed recirculated groundwater discharge in Group P samples, it was likely that these bacteria received from the seawater. An indoor mesocosm experiment with natural plankton communities from the western Baltic Sea revealed that *Glaciecola* sp. was the single most abundant taxon during the phytoplankton peak in the cold water (Scheibner et al., 2014). High abundance of *Glaciecola* cells has also been observed in temperate North Sea during a spring bloom (Teeling et al., 2012). The considerable presence of Genus *Glaciecola* during the blooming phase of the diatoms in different studied regions suggested its significant roles in consuming dissolved organic carbon released by phytoplankton. YSGW7 contained 13.5% *Glaciecola* related sequences, indicated that SGD was significant source of the carbon flux to the coastal water. Meanwhile, *Gammaproteobacteria* was observed as dominated bacterial group during the phytoplankton peak (Teeling et al., 2012; Scheibner et al., 2014), the coexistence of *Gammaproteobacteria* and *Cyanobacteria* in our studied porewater sample was probably intensified contributions of the nutrient and DOC by SGD to the occurring of macroalgae bloom in the coastal areas.

Several studies of bacterial communities on the surface of marine macroalgae have shown the distinctive compositions of planctomycetes could be observed in association with different marine macroalgae (Lage and Bondoso, 2011; Bondoso et al., 2014). The second most abundant OTU 1051 in YSPW 11 was closely related to *Planctomycetes Rhodopirellula Baltic* strain SH 1^T^, this strain was also frequently found in epiphytic communities of many macroalgae in a widespread geographic locations (Lage and Bondoso, 2011). The genome of *Rhodopirellula baltic* strain SH 1^T^ contains a large number of sulfatases, which are known to be involved in carbon recycling of complex sulfated heteropolysaccharides (Glöckner et al., 2003). The ability of *Planctomycetes* to degrade sulfatated heteropolysaccharides provides us the efficient pathway to obtain polysaccharides with lower molecular weights in porewaters, which were proved to possess important pharmacological activities such as antioxidant activities (Qi et al., 2005; Costa et al., 2010; Li et al., 2013).

#### The putative ecological role of genus *Limnohabitans* in the fresh well water

*Betaproteobacteria* and *Actinobacteria* constituted the two major groups in four fresh well water samples, in agreement with the finding that a few phylogenetic clusters are dominant in a variety of freshwater ecosystems (Allgaier and Grossart, 2006; Newton et al., 2011). Our correlation analysis confirmed *Betaproteobacteria* favored lower salinity condition (Figure 7).

Members of Genus *Limnohabitans* within *Betaproteobacteria* were frequently predominant in freshwater bacterioplankton communities (Šimek et al., 2010b). We reported that Bacteria of the genus *Limnohabitans* were the major group in the fresh well samples (Figure 3). *Limnohabitans* spp. are the well-studied opportunistic bacterial groups (Šimek et al., 2005), with fast living generation time, specialization on low molecular weight dissolved organic matter, and highly vulnerability to size selective predation (Salcher, 2013). Autochthonous algal-derived organic matters and products of the photolysis of dissolved materials are two main groups of substrates for the growth of members of Genus *Limnohabitans* (Šimek et al., 2011). Genus *Limnohabitants* was identified as an important player in in carbon flow to higher trophic levels since they can be consumed by food for heterotrophic nanoflagellates (Šimek et al., 2013). More recent study showed *Limnohabitans* Species Strains Rim28 and Rim47 had a great metabolic versatility, including photosynthesis, autotrophic carbon fixation, ammonium oxidation and sulfur oxidization (Zeng et al., 2012b). The higher nitrate concentrations in YSGW3 and YSGW 11 was in good agreement with the most abundant sequences affiliated with *Limnohabitans* sp. Rim 28, which may serve as ammonium oxidizer in these two samples. Fresh well waters provide another potential sources for both nitrogen and carbon flux to the coastal waters.

#### Bacterial candidate for bioremediation in well waters

Fresh well sample YSGW 4 is located in a public park in Shandong province, garbage such as mosquito and discarded beverage bottles in the well were observed during the sampling time. Compared to the other three fresh water samples, YSGW 4 exhibited relatively lower nitrate and higher DOC concentrations. The co-occurrence of sequences closely related to *Actinobacteria Rhodoluna lacicola* strain MWH-EgelM2-3.2 (FJ545223), *Flavobacterium granuli* strain kw05 (NR_041052), and uncultured *Flectobacillus* clone ZS-2-379 (FN668111) (Figure 4). The presence of actinorhodopsins in *Rhodoluna lacicola* strain MWH-EgelM2-3.2 suggested this strain to be photoheterotrophic bacteria, surviving under low nutrient and energy conditions (Sharma et al., 2008). *Flavobacterium granuli* was isolated from granules used in a wastewater treatment plant (Aslam et al., 2005); Members of genus *Flectobacillus* usually act as the direct competitors *in situ* (Šimek et al., 2010a). The characteristics of these three groups indicated the hostile environment of YSGW 4 sampling site. The most abundant sequences in YSGW 4 were affiliated with *C. testoteroni* strain CNB-2. The complete genome of strain CNB-2 revealed its genetic versatility adaption to diverse habitats, based on its ability to metabolize a variety of compounds, such as carboxylic acids and aromatic compounds, utilize nitrate and ammonia as nitrogen source, resistant to drug and heavy metals (Ma et al., 2009). *C. testosteroni* strain CNB-1 has been used successfully rhizoremediation of 4-chloronitrobenzene (CNB) polluted soil (Liu et al., 2007). Other *Comamonas* spp. were also used for environmental applications (Tobajas et al., 2012; Wu et al., 2015). The high abundance of *C. testosteroni* related sequences in our samples indicated that this species could be selected as potential microbes for bioremediation in the highly polluted fresh well waters before flowing into coastal water.

#### Linking bacterial community with environmental variables

Based on our analysis of relationship between bacterial taxonomic groups and contexture environmental parameters, we found *Betaproteobacteria* in well water had highly negative correlation with ammonium, but had positive correlation with nitrate; in contrast, *Cyanobacteria* in porewater had highly positively correlation with ammonium. These results supported the possibilities that fresh well water and brackish porewater may provide nitrate and ammonium respectively. Only YSPW 2 contained more than 1% sequences within six rare bacterial taxonomic groups (Figure 7), which had highly positively correlation with nitrite, showing these groups in YSPW2 had the abilities to cycle nitrogen.

Four well water samples were collected from three different eco-environments impacted by heavy human activities (Table 1). We detected less diverse bacterial groups in the well water samples than those in the porewater samples (Table 2), suggesting the stressful conditions in the well water due to anthropogenic activities. *Comamona*s spp. and *Limnohabitans* spp. were dominated in fresh well samples, but were almost absent from the brackish porewater samples, which indicates these groups favored lower salinity environment. Distinctive bacterial patterns were detected among the three porewater samples collected from the different tourist beaches, for example, six rare bacterial taxonomic groups were mainly found in YSGW 2, which may have been due to niche selections.

## Conclusion

Our study documented bacterial community structures in the both fresh well water and brackish porewater along the coasts of the Yellow Sea. We provided perspective into the correlations between representative bacterial taxonomic groups and environmental parameters, fresh well water and brackish porewater provided the different nitrogen sources to the coastal water. Potential key bacterial groups such as *Comamonas testosteroni* may be excellent candidates for bioremediation of the natural pollutants in different niches. Further studies will explore the shifts of indigenous microbial community in *in-situ* bioremediation experiments in the submarine groundwater.

## Author contributions

JD designed the experiments; JL collected the samples; QY and JL performed the experiments and analyzed the data; QY, JL, JD, and JZ wrote the manuscript.

### Conflict of interest statement

The authors declare that the research was conducted in the absence of any commercial or financial relationships that could be construed as a potential conflict of interest.
