# Supplementary material for: Bacterial Diversity in Submarine Groundwater along the Coasts of the Yellow Sea
Source: Front Microbiol. 2016 Jan 8;6:1519. doi: 10.3389/fmicb.2015.01519 (PMC4705239; doi:10.3389/fmicb.2015.01519)
Supplement: Supplementary file 1 [file Table1.docx]

Table S1 Representative taxonomic groups in the studied samples

|  |  | YSGW1 | YSGW3 | YDGW4 | YSGW  11 | YSPW2 | YSPW7 | YSPW  11 |
| --- | --- | --- | --- | --- | --- | --- | --- | --- |
| Abundant  Taxonomic group | *Betaproteobacteria*  (31296) | **66.21** | **38.70** | **42.65** | **70.58** | 8.01 | 0.52 | 1.14 |
|  | *Gammaproteobacteria*  (9080) | **5.68** | **12.94** | 0.06 | **1.27** | **18.42** | **31.75** | **11.97** |
|  | *Flavabacterria*  (8758) | **13.04** | 0.38 | **26.86** | 0.08 | **3.71** | **6.00** | **16.89** |
|  | *Actinobacteria*  (8693) | 0.25 | **27.23** | **16.68** | **11.57** | **5.95** | **2.55** | **5.94** |
|  | *Cyanobacteria*  (7199) | 0.20 | 0 | 0.95 | 0.18 | **1.60** | **32.81** | **22.06** |
|  | *Alphaproteobacteria*  (7165) | **3.15** | **9.17** | **2.08** | **4.81** | **10.64** | **19.31** | **11.68** |
|  | *Cytophagia*  (3140) | 0.15 | 1.73 | **10.24** | **4.38** | **1.81** | **1.07** | **4.25** |
|  | *Planctomycetes*  *(2297)* | 0.64 | 1.19 | 0.01 | **3.65** | **17.23** | **1.66** | **13.37** |
|  | *OP3*  (628) | **3.03** | **1.50** | 0 | 0.16 | 0.01 | 0 | 0.01 |
| rare  Taxonomic group | *Acidobacteria*  (1149) | 0.44 | 0.29 | 0 | 0.02 | **12.01** | 0.43 | 0.70 |
|  | *Nitrospirae*  （532） | 0.59 | 0.28 | 0.00 | 0.01 | **5.31** | 0.05 | 0.11 |
|  | *Chloroflexi*  (161) | 0.04 | 0 | 0 | 0.02 | **1.61** | 0.10 | 0.14 |
|  | *Gemmatimonadetes*  (161) | 0.01 | 0 | 0 | 0 | **1.76** | 0.09 | 0.11 |
|  | *Verrucomicrobia*  (157) | 0.01 | 0.03 | 0 | 0.05 | **1.17** | 0.17 | 0.25 |
|  | W3  (106) | 0 | 0 | 0 | 0 | **1.32** | 0.02 | 0.03 |
|  | *Firmicutes*  (371) | 0.20 | **1.73** | 0.05 | 0.21 | 0.08 | 0.42 | 0.40 |
|  | *TM7*  (252) | 0 | 0 | 0 | 0 | 0.27 | 0.22 | **1.40** |
|  | *Armatimonadetes*  (202) | 0.01 | 0 | 0 | **1.21** | 0 | 0 | 0.01 |

Sequences abundance (>1%) of each phylum is shown in bold.
